# Gestational Diabetes and Preeclampsia in Association with Air Pollution at Levels below Current Air Quality Guidelines

**DOI:** 10.1289/ehp.1205736

**Published:** 2013-01-16

**Authors:** Ebba Malmqvist, Kristina Jakobsson, Håkan Tinnerberg, Anna Rignell-Hydbom, Lars Rylander

**Affiliations:** Division of Occupational and Environmental Medicine, Lund University, Lund, Sweden

**Keywords:** air pollution, gestational complications, gestational diabetes, NO_x_, preeclampsia, traffic

## Abstract

Background: Several studies have estimated associations between air pollution and birth outcomes, but few have evaluated potential effects on pregnancy complications.

Objective: We investigated whether low-level exposure to air pollution is associated with gestational diabetes and preeclampsia.

Methods: High-quality registry information on 81,110 singleton pregnancy outcomes in southern Sweden during 1999–2005 was linked to individual-level exposure estimates with high spatial resolution. Modeled exposure to nitrogen oxides (NO_x_), expressed as mean concentrations per trimester, and proximity to roads of different traffic densities were used as proxy indicators of exposure to combustion-related air pollution. The data were analyzed by logistic regression, with and without adjusting for potential confounders.

Results: The prevalence of gestational diabetes increased with each NO_x_ quartile, with an adjusted odds ratio (OR) of 1.69 (95% CI: 1.41, 2.03) for the highest (> 22.7 µg/m^3^) compared with the lowest quartile (2.5–8.9 µg/m^3^) of exposure during the second trimester. The adjusted OR for acquiring preeclampsia after exposure during the third trimester was 1.51 (1.32, 1.73) in the highest quartile of NO_x_ compared with the lowest. Both outcomes were associated with high traffic density, but ORs were significant for gestational diabetes only.

Conclusion: NO_x_ exposure during pregnancy was associated with gestational diabetes and preeclampsia in an area with air pollution levels below current air quality guidelines.

Air pollution is a heterogeneous mixture of gases and solid particles in the air, each component having its own potential effect on the human body. There is increasing evidence that air pollution may cause systemic inflammation and elevated blood pressure (Brook 2008; Brook and Rajagopalan 2009). Several studies have explored the association between air pollution and negative birth outcomes (Bobak 2000; Liu et al. 2003; Malmqvist et al. 2011; Slama et al. 2009; Wilhelm and Ritz 2003), but few studies have studied the effect of air pollution on pregnancy complications. Preeclampsia and other pregnancy complications have been identified as priority research areas relating to air pollution and reproductive effects (Woodruff et al. 2009). Adverse maternal outcomes are of relevance per se, but may also provide insights into the mechanisms behind negative birth outcomes.

Previous studies have reported associations between air pollution and type 2 diabetes, and it has been proposed that systemic inflammation may be the underlying biological mechanism (Brook et al. 2008; Krämer et al. 2010; Puett et al. 2010). Pregnancy is a condition with an increased susceptibility to diabetogenesis (Galtier 2010); consequently, pregnant women might be a group susceptible to potential air pollution effects on the incidence of diabetes. To our knowledge there has been only one previous study of air pollution and gestational diabetes, which reported no association (van den Hooven et al. 2009).

Preeclampsia causes maternal mortality, morbidity, perinatal death, intrauterine growth restriction, and preterm birth (Sibai et al. 2005). Several recent studies have reported positive associations between preeclampsia and air pollutants including nitrogen oxides (NO_x_), nitrogen monoxide (NO), nitrogen dioxide (NO_2_), carbon monoxide (CO), ozone (O_3_), particulate matter < 2.5 μm in aerodynamic diameter (PM_2.5_), and particulate matter < 10 μm in aerodynamic diameter (PM_10_) (Lee et al. 2012; Wu et al. 2009, 2011), but others have reported no association with PM_2.5_ or CO (Rudra et al. 2011) and inconclusive findings for PM_10_ and NO_2_ (van den Hooven et al. 2011). Two studies have reported positive associations between gestational hypertension (a risk factor and early symptom of preeclampsia) and air pollutants [PM_10_ and PM_2.5_ (Vinikoor-Imler et al. 2012) and NO_2_ and PM_10_ (van den Hooven et al. 2011)].

Etiologic mechanisms leading to gestational diabetes and preeclampsia are uncertain, but systemic inflammation may be a contributing factor (Sibai et al. 2005; Wellen and Hotamisligil 2005). Both experimental and observational evidence indicates that exposure to air pollution, particularly ultrafine particles, induces oxidative stress and consequently inflammation (reviewed by Terzano et al. 2010).

In previous studies, levels of NO_x_ have been shown to correspond well with levels of combustion-related particulate matter < 1 μm in aerodynamic diameter (PM_1_), or even for combustion-related ultrafine particulate matter (< 0.1 μm; PM_0.1_) (Arhami et al. 2009; Ketzel et al. 2004; Marconi et al. 2007). Ambient NO_2_ concentrations in Scania, Sweden, the site of the present study, are generally well below current World Health Organization (WHO) air quality guidelines (NO_2_ annual average < 40 µg/m^3^; WHO 2006). Exposure data of high spatial resolution are readily available from a recent cohort study of > 80,000 children born in Scania during 1999–2005; this study explored the relationship between maternal exposure to air pollution and birth outcomes (Malmqvist et al. 2011). The aim of the present study was to assess maternal morbidity in this cohort. The Swedish Medical Birth Registry (National Board for Health and Welfare, Stockholm, Sweden) contains information from maternal health care records on relevant confounders, and almost all pregnant women (98–99%) attend the tax-subsidized antenatal health care system (Socialstyrelsen 2002), where a screening program for gestational diabetes has been operating since 1995. Thus, information on maternal outcomes and exposures in the total population was available, which is unique.

## Materials and Methods

*Study area*. The study was carried out in Scania, a county in southern Sweden. Sweden’s third largest city (Malmö; ~ 260,000 inhabitants) is located in Scania, and the county is densely populated compared with the rest of the country, with approximately 1.2 million people living within 11,000 km^2^. Because of its proximity to the European continent and the Danish capital, Copenhagen, a great deal of cargo is transported to, from, and through this region by road, rail, and water, resulting in high levels of emissions relative to other parts of Sweden. Although the air pollution levels vary considerably within the county, they are generally below the present WHO air quality guidelines for NO_2_, PM_10_, and O_3_ (Sjöberg et al. 2006; WHO 2006).

*Study population*. The Swedish Medical Birth Registry includes almost all (98–99%) infants born in Sweden (Socialstyrelsen 2002). The present study included 81,110 women who had singleton deliveries during 1999–2005. The study population has previously been described in detail (Malmqvist et al. 2011). This study was approved by the Lund University Ethical Committee. No formal informed consent was required, but the study was advertised in the local newspaper and women could request not to be included in it, though none did.

*Outcome measurements*. Gestational diabetes. In Scania, an oral glucose tolerance test is routinely performed during pregnancy week 28 to screen for gestational diabetes. The test is offered to all pregnant women and has a participation rate of approximately 93% (Anderberg et al. 2007). Women with a first-degree family history of diabetes, or a history of gestational diabetes in a previous pregnancy, are additionally screened during week 12. Gestational diabetes is diagnosed based on plasma glucose > 10 mmol/L 2 hr after oral administration of 75 g of glucose. If results indicate impaired glucose tolerance (plasma glucose 8.6–9.9 mmol/L), the test is repeated within a week (Anderberg et al. 2010). If a diagnosis of gestational diabetes was not indicated in the Swedish Medical Birth Registry, women were presumed not to have gestational diabetes. There were 1,599 cases of gestational diabetes registered.

Preeclampsia. Blood pressure and proteinuria are routinely measured at each visit to the maternal health care units, and preeclampsia is diagnosed according to the *International Classification of Diseases, 10th Revision* (WHO 1993; codes P07.0–P07.3) based on systolic blood pressure ≥ 140 mmHg and/or diastolic blood pressure ≥ 90 mmHg (measured twice with at least 6 hr between) and proteinuria > 0.3 g/day after 20 weeks of gestation. The diagnostic criterion for severe preeclampsia is blood pressure ≥ 160/110 mmHg or proteinuria ≥ 5 g/day. Altogether, there were 2,370 cases of preeclampsia, including 1,799 classified as mild and 571 as severe. Women without a diagnosis of preeclampsia in the Swedish Medical Birth Registry were presumed not to have preeclampsia.

*Exposure assessments*. Modeled NO_x_ exposure. We acquired information on NO_x_ in Scania from an emission database (EDB) with information on approximately 24,000 sources (Stroh et al. 2005) including line sources (road traffic, shipping, and railroads), point sources (industries and larger energy and heat producers), and area sources (aviation, small-scale heating, and construction machinery). Emissions from eastern Denmark, which are quite high in Scania due to prevailing westerly winds, were also included (Kristensson et al. 2008). For dispersion calculations, the EDB was combined with AERMOD, a modified Gaussian flat two-dimensional dispersion model (U.S. Environmental Protection Agency 2004) that incorporates meteorological data (Stroh et al. 2005). Modeled concentrations with a spatial resolution of 100 × 100 m were highly correlated with façade concentrations measured over 1-week periods (Spearman’s correlation coefficient = 0.8, *n* = 142) (Stroh et al. 2012). To account for long-range transboundary air pollution (in addition to the emissions from Eastern Denmark) we added a background level of 2.5 µg/m^3^ to the model, which corresponds to the yearly mean value obtained from a local remote background monitor station.

For the present study, concentrations of NO_x_ were modeled as hourly means with a spatial resolution of 500 × 500 m. Hourly means were aggregated into monthly and trimester means for each pregnancy (gestation months 1–3, 4–6, and 7–delivery). Different spatial resolutions have been studied, and a resolution of 500 × 500 m was considered optimal when calculating monthly means in this study area, because temporal aggregation allowed lower spatial resolution without significant loss of detail (Stroh et al. 2007). Concentrations were calculated at the women’s geocoded residence.

Because of migration in and out of the county, NO_x_ exposures could not be estimated for the first, second, and third trimesters for 5,201, 4,756, and 4,585 women, respectively. Women were excluded from trimester-specific analyses when trimester-specific data were missing, but were included in all other analyses with available exposure data.

The average level of modeled NO_x_ exposure decreased from 18.2 µg/m^3^ in 1999 to 13.3 µg/m^3^ in 2005, with an average of 16.4 µg/m^3^ over the entire study period. NO_x_ was categorized into quartiles based on the distribution of individual exposures during the first trimester [reference, 2.5–8.9 µg/m^3^ (mean = 6.8 µg/m^3^); 9.0–14.1 µg/m^3^ (mean = 11.4 µg/m^3^); 14.2–22.6 µg/m^3^ (mean = 18.2 µg/m^3^); ≥ 22.7 µg/m^3^ (mean = 29.6 µg/m^3^)].

*Traffic density*. Road traffic data were obtained from the Swedish National Road Database (Vägverket 2012) and used to calculate the distance between the geocoded location of each mother’s residence during the first trimester and the road with the heaviest traffic density (annual 24-hr mean value) within a 200-m radius. Mothers who migrated into Scania after the first trimester were excluded from analyses of traffic density (*n* = 5,536). Traffic density was categorized as no road within 200 m (reference category), road with < 2 cars/min, road with 2–5 cars/min, road with 6–10 cars/min, or road with > 10 cars/min. These categories were chosen to correspond with previous studies in this area (Lindgren et al. 2009; Malmqvist et al. 2011).

*Covariates*. We adjusted analyses of preeclampsia for major risk factors including prepregnancy body mass index (BMI), smoking habits at first antenatal visit, ethnicity (based on country of birth), parity, type 1 diabetes, gestational diabetes, and maternal age (Schneider et al. 2012; Sibai et al. 1997, 2005), which were available from the Swedish Medical Birth Registry for the women in the study. These factors, except smoking, are also relevant risk factors for gestational diabetes, and were therefore included in models of that outcome as well (Galtier 2010; Schneider et al. 2012). These covariates were categorized as shown in Table 1, including categories for missing data on pre-pregnancy BMI and smoking. To account for calendar year effects, we also adjusted for year of delivery (1999–2005). To account for multiple pregnancies per woman, we conducted restricted analyses including only first pregnancies.

**Table 1:** Description of study subjects [*n* (%)]

Characteristic	Preeclampsia (n = 2,323)	Gestational diabetes (n = 1,599)	All (n = 81,110)
Maternal age (years)
< 25	357 (15.4)	141 (8.8)	11,708 (14.4)
25–30	745 (32.1)	409 (25.6)	26,256 (32.4)
30–35	767 (33.0)	544 (34.0)	28,401 (35.0)
≥ 35	454 (19.5)	505 (31.6)	14,745 (18.2)
Country of origin
Sweden	1,877 (84.6)	972 (63.0)	62,128 (76.6)
Other Nordic	32 (1.4)	19 (1.2)	1,564 (1.9)
Other Western	22 (1.0)	17 (1.1)	1,111 (1.4)
Eastern Europe	118 (5.3)	118 (7.6)	5,576 (6.9)
Africa Sub Sahara	27 (1.2)	48 (3.1)	884 (1.1)
Middle East/North Africa	95 (4.3)	270 (17.5)	5,754 (7.1)
Asia	25 (1.1)	84 (5.4)	1,858 (2.3)
South/Central America	23 (1.0)	16 (1.0)	738 (0.9)
Parity
1	1,603 (69.0)	674 (42.2)	38,365 (47.3)
2	464 (20.0)	485 (30.3)	26,681 (32.9)
≥ 3	255 (11.0)	440 (27.5)	15,418 (19.0)
BMI
< 18.5	37 (1.6)	23 (1.4)	2,944 (3.6)
18.5–24.9	640 (27.6)	536 (33.5)	31,629 (39.0)
25–29.9	598 (25.7)	432 (27.0)	17,836 (22.0)
≥ 30	477 (20.5)	375 (23.5)	7,852 (9.7)
Missing	571 (24.6)	233 (14.6)	20,849 (25.7)
Smoking (cigarettes/day)
0	1,902 (81.9)	1,264 (79.0)	66,086 (81.5)
1–9	128 (5.5)	113 (7.1)	6,444 (7.9)
> 10	44 (1.9)	54 (3.4)	2,948 (3.6)
Missing	249 (10.7)	168 (10.5)	5,632 (6.9)
Diabetes type 1	53 (2.3)	301 (18.8)	406 (0.5)
Chronic hypertension	110 (4.7)	28 (1.8)	352 (0.4)
Changed residency	325 (13.3)	160 (10.0)	9,157 (11.3)
Birth year
1999	281 (12.1)	144 (9.0)	10,593 (13.1)
2000	283 (12.2)	182 (11.4)	11,018 (13.6)
2001	287 (12.4)	268 (16.8)	11,107 (13.7)
2002	310 (13.3)	208 (13.0)	11,720 (14.4)
2003	316 (13.6)	277 (17.3)	11,473 (14.1)
2004	355 (15.3)	295 (18.4)	12,449 (15.3)
2005	491 (21.1)	225 (14.1)	12,750 (15.7)
Gestational diabetes	119 (5.1)		1,599 (2.0)

No information was available on other risk factors for gestational diabetes—previous gestational diabetes, family history of type 2 diabetes, polycystic ovary syndrome, low own birth weight, and low physical activity.

*Database linkage*. Each resident of Sweden has a unique 10-digit personal identification code that can be linked to the center coordinate of her or his location of residence (updated yearly). A woman could have multiple locations and thus different trimester-specific exposure if she moved residence during pregnancy. However, we lacked information on exact timing of the move, and to account for this we conducted restricted analyses with women who had not moved during pregnancy. Residential coordinates were linked to modeled exposure and traffic data by using a geographical information system, and each assessed individual exposure was subsequently linked to information from the Swedish Medical Birth Registry, with the aid of the personal identification code.

*Statistics*. Gestational diabetes and preeclampsia were examined as dichotomous outcomes in logistic regression models, using IBM SPSS Statistics, version 20 (IBM Inc., Chicago, IL, USA). In addition to analyses of all preeclampsia cases, we conducted separate analyses restricted to mild and severe preeclampsia cases, respectively. Women with type 1 diabetes (*n* = 406) were excluded from analyses of gestational diabetes, and women with chronic hypertension (*n* = 352) were excluded from analyses of preeclampsia, thus avoiding potential misclassification of cases as noncases.

For the gestational diabetes analyses we estimated associations with NO_x_ exposure during the first or second trimester only—before the screening in week 28. Associations with NO_x_ exposure during all three trimesters were estimated for the preeclampsia analyses.

Associations between NO_x_, traffic density and gestational diabetes were estimated for all women without adjustment and with adjustment for maternal age, parity, BMI, calendar year, and country of origin. In addition we estimated crude and adjusted odds ratios (ORs) based on analyses restricted to primiparae, to women who did not move residence during pregnancy, and to women who were born in Nordic countries.

Associations between NO_x_, traffic density, and preeclampsia were estimated for all women without adjustment and with adjustment for maternal age, parity, smoking, BMI, gestational diabetes, type 1 diabetes, country of origin, and calendar year. As for analyses of gestational diabetes, we also ran analyses restricted to primiparae, women who did not move residence during pregnancy, and women born in Nordic countries. In addition, we repeated all analyses after excluding women diagnosed with severe preeclampsia, and after excluding women diagnosed with mild preeclampsia from the analyses.

Because NO_x_ levels vary between urban and nonurban areas, associations may be confounded by unmeasured environmental and socioeconomic risk factors that also differ between urban and non-urban areas. Therefore, we subdivided the cohort into urban women living in the two main cities, Malmö and Helsingborg, and non-urban women (all others) and estimated separate associations between the outcomes and NO_x_ above versus below the average level for each group (21.2 µg/m^3^ for the urban group, and 8.9 µg/m^3^ for the non-urban group). These analyses were restricted to Nordic-born women to reduce potential confounding by other unmeasured factors related to ethnicity. Analyses were performed with fully adjusted models for all outcomes.

## Results

*Covariates and potential confounding*. Women in the highest NO_x_ quartile were predominantly primiparae (55% fourth quartile compared with 40% first quartile) and had non-Nordic origin (35% fourth quartile compared with 8% first quartile) [see Supplemental Material, Table S1 (http://dx.doi.org/10.1289/ehp.1205736)]. In contrast, > 90% of women within the lowest quartile were of Swedish origin. There were no differences in smoking between quartiles. There was a higher prevalence of overweight/obese (BMI ≥ 25) in the lowest quartile of NO_x_ (37%) compared with the highest (29%). Notably, women giving birth during the first 4 years compared with the last 2 years of the study period dominated in the highest NO_x_ category.

*Gestational diabetes*. High parity, high BMI, age *≥* 35 years, type 1 diabetes, and chronic hypertension were more frequent features in women with gestational diabetes than the cohort as a whole (Table 1). Diabetic women were more likely than the cohort as a whole to have missing information on smoking (11% vs. 7%). A higher proportion of diabetic women came from the Middle East/North Africa, sub-Saharan Africa, and Asia than the cohort as a whole.

Crude and adjusted ORs for gestational diabetes increased monotonically with increasing NO_x_ exposure levels during the second trimester for second-, third-, and fourth-quartile crude ORs [1.28 (95% CI: 1.07, 1.54); 1.84 (95% CI: 1.56, 2.18), and 1.98 (95% CI: 1.68, 2.35), respectively], and for second-, third-, and fourth-quartile adjusted ORs [1.19 (95% CI: 0.99–1.44); 1.52 (95% CI 1.28, 1.82); and 1.69 (95% CI: 1.41, 2.03), respectively] (Table 2). Corresponding adjusted ORs from restricted analyses were 1.80 (95% CI: 1.49, 2.19) for mothers who did not move residence during pregnancy, 1.56 (95% CI: 1.24, 1.95) for Nordic-born mothers, and 1.53 (95% CI: 1.14, 2.05) for first-time mothers [see Supplemental Material, Table S2 (http://dx.doi.org/10.1289/ehp.1205736)]. ORs for NO_x_ exposure during the first trimester were similar to those for exposure during the second trimester (see Supplemental Material, Table S3).

**Table 2:** ORs for gestational diabetes in relation to NO_x_ (µg/m^3^) and traffic density (vehicles/min).

Exposure	Crude OR (95% CI)	Adjusted OR (95% CI)
NOx quartile		
Q1 = 2.5–8.9	1.00 (reference)	1.00 (reference)
Q2 = 9.0–14.1	1.28 (1.07, 1.54)	1.19 (0.99, 1.44)
Q3 = 14.2–22.6	1.84 (1.56, 2.18)	1.52 (1.28, 1.82)
Q4 = > 22.7	1.98 (1.68, 2.35)	1.69 (1.41, 2.03)
Traffic density within 200 m		
No road	1.00 (reference)	1.00 (reference)
< 2	0.89 (0.75, 1.06)	0.93 (0.78, 1.12)
2–5	1.04 (0.88, 1.23)	0.96 (0.81, 1.14)
5–10	1.53 (1.27, 1.84)	1.18 (0.97, 1.43)
> 10	1.50 (1.24, 1.82)	1.23 (1.01, 1.51)
Gestational diabetes model adjusted for parity, BMI, maternal age, calendar year, and country of origin and exposure for second trimester.		

ORs for traffic density at the nearest road within 200 m suggested an association with residences in areas with > 10 vehicles/min (adjusted OR = 1.23; 95% CI: 1.01, 1.51) compared with no road within 200 m (Table 2). The association was similar when restricted to nonmovers, but ORs were close to the null when the analysis was restricted to Nordic-born mothers and primiparae [see Supplemental Material, Table S2 (http://dx.doi.org/10.1289/ehp.1205736)].

Among Nordic-born women, the prevalence of gestational diabetes was higher among women living in an urban versus non-urban area (adjusted OR = 1.20; 95% CI: 0.99, 1.46). In addition, exposure to NO_x_ above the average value was positively associated with gestational diabetes among both urban (adjusted OR = 1.15; 95% CI: 0.98, 1.34 for second-trimester exposure > 21.2 µg/m^3^) and non-urban mothers (adjusted OR = 1.37; 95% CI: 1.18, 1.60 for second-trimester exposure > 8.9 µg/m^3^) [see Supplemental Material, Table S4 (http://dx.doi.org/10.1289/ehp.1205736)].

Estimated associations between gestational diabetes and the highest versus lowest quartile of second-trimester NO_x_ exposure among Nordic-born mothers (adjusted OR = 1.56; 95% CI: 1.24, 1.95) was comparable to the estimated effect of being *≥* 35 years of age (adjusted OR = 2.32; 95% CI: 1.89, 2.84 compared with age < 25 years) and of being overweight (adjusted OR = 1.87; 95% CI: 1.55, 2.25 for BMI 25–29.9), but was weaker than the estimated effect of being obese (adjusted OR = 4.05; 95% CI: 3.34, 4.91 for BMI *≥* 30) [see Supplemental Material, Table S5 (http://dx.doi.org/10.1289/ehp.1205736)].

*Preeclampsia*. Women with preeclampsia were more likely to be primiparous, of Swedish origin, and obese (BMI *≥* 30) but less likely to be heavy smokers than the cohort as a whole (Table 1). Women with preeclampsia also were more likely to have missing information on BMI and smoking than the cohort as a whole.

Crude and adjusted ORs for preeclampsia increased with increasing quartiles of third-trimester NO_x_ exposures [adjusted ORs for the second, third, and fourth quartiles of 1.28 (95% CI: 1.13, 1.46), 1.33 (95% CI: 1.17, 1.52), and 1.51 (95% CI: 1.32, 1.71, respectively] (Table 3). Adjusted ORs for the highest versus lowest quartile from restricted analyses were 1.48 (95% CI: 1.27, 1.70) for mothers who did not move residence during pregnancy; 1.51 (95% CI: 1.31, 1.74) for Nordic-born mothers; and 1.61 (95% CI: 1.37, 1.90) for first-time mothers [see Supplemental Material, Table S6 (http://dx.doi.org/10.1289/ehp.1205736)].

**Table 3:** ORs for preeclampsia in relation to NO_x_ (µg/m^3^) and traffic density (vehicles/min).

Outcome/exposure	Crude OR (95% CI)	Adjusted OR (95% CI)
Preeclampsia/NOx quartile		
Q1 = 2.5–8.9	1.00 (reference)	1.00 (reference)
Q2 = 9.0–14.1	1.21 (1.07, 1.38)	1.28 (1.13, 1.46)
Q3 = 14.2–22.6	1.26 (1.11, 1.43)	1.33 (1.17, 1.52)
Q4 = > 22.7	1.34 (1.18, 1.52)	1.51 (1.32, 1.73)
Preeclampsia mild/NOx quartile		
Q1 = 2.5–8.9	1.00 (reference)	1.00 (reference)
Q2 = 9.0–14.1	1.29 (1.12, 1.49)	1.38 (1.19, 1.60)
Q3 = 14.2–22.6	1.32 (1.14, 1.52)	1.42 (1.23, 1.65)
Q4 = > 22.7	1.32 (1.14, 1.53)	1.51 (1.29, 1.77)
Preeclampsia severe/NOx quartile		
Q1 = 2.5–8.9	1.00 (reference)	1.00 (reference)
Q2 = 9.0–14.1	0.97 (0.74, 1.26)	0.96 (0.72, 1.28)
Q3 = 14.2–22.6	1.03 (0.79, 1.34)	0.99 (0.74, 1.32)
Q4 = > 22.7	1.42 (1.10, 1.83)	1.48 (1.12, 1.95)
Preeclampsia/traffic density within 200 m		
No road	1.00 (reference)	1.00 (reference)
< 2	1.14 (1.00, 1.29)	1.12 (0.98, 1.28)
2–5	1.09 (0.96, 1.24)	1.04 (0.91, 1.19)
5–10	1.14 (0.98, 1.32)	1.14 (0.97, 1.34)
> 10	1.19 (1.02,1.40	1.10 (0.94,1.30)
Preeclampsia mild/traffic density within 200 m		
No road	1.00 (reference)	1.00 (reference)
< 2	1.09 (0.95, 1.27)	1.06 (0.91, 1.23)
2–5	1.06 (0.92, 1.23)	1.02 (0.88, 1.19)
5–10	1.15 (0.97, 1.37)	1.13 (0.95, 1.36)
> 10	1.13 (0.95, 1.36)	1.04 (0.86, 1.26)
Preeclampsia severe/traffic density within 200 m		
No road	1.00 (reference)	1.00 (reference)
< 2	1.27 (0.97, 1.66)	1.37 (1.03, 1.83)
2–5	1.18 (0.90, 1.54)	1.13 (0.84, 1.53)
5–10	0.99 (0.71, 1.40)	1.08 (0.75, 1.56)
> 10	1.27 (0.92, 1.77)	1.24 (0.86, 1.77)
Preeclampsia models adjusted for gestational diabetes, type 1 diabetes, smoking, parity, BMI, country of origin, calendar year, and maternal age.		

ORs for mild preeclampsia (*n* = 1,799) were similar to those for all cases, whereas severe preeclampsia was only associated with the highest quartile of exposure (adjusted OR = 1.48; 95% CI: 1.12, 1.95) (Table 3). ORs from models restricted to nonmovers, Nordic-born women, and first-time mothers were similar to those for all cases combined [see Supplemental Material, Table S6 (http://dx.doi.org/10.1289/ehp.1205736)].

Estimated associations for first- and second-trimester NO_x_ exposures were similar to ORs for third-trimester NO_x_ exposure for preeclampsia, mild preeclampsia, and severe preeclampsia [see Supplemental Material, Table S7 (http://dx.doi.org/10.1289/ehp.1205736)]. No clear associations between traffic density at the nearest road within 200 m and prevalence of preeclampsia were observed (Table 3; see also Supplemental Material, Table S6).

Among Nordic-born women, preeclampsia, mild preeclampsia, and severe preeclampsia all were significantly increased among urban versus non-urban women (e.g., adjusted OR for all preeclampsia = 1.40; 95% CI: 1.27, 1.54), but all three outcomes also were positively associated with NO_x_ exposure above average levels among women living in both urban and non-urban areas [adjusted ORs for all preeclampsia of 1.27 (95% CI: 1.13, 1.44) and 1.37 (95% CI: 1.24, 1.51), respectively] [see Supplemental Material, Table S4 (http://dx.doi.org/10.1289/ehp.1205736)].

Estimated associations of the highest versus lowest quartile of NO_x_ exposure with preeclampsia among Nordic-born women (e.g., adjusted OR for all preeclampsia = 1.51; 95% CI: 1.31, 1.74) were comparable to estimated effects of being > 35 years of age (adjusted OR = 1.20; 95% CI: 1.03, 1.39), of having gestational diabetes (OR = 2.70; 95% CI: 1.76, 4.15), and of being overweight (OR = 1.85; 95% CI: 1.64, 2.09 for BMI 25–29.9), but less than the estimated effect of being obese (adjusted OR = 3.75; 95% CI: 3.28, 4.28 for BMI *≥* 30) [see Supplemental Material, Table S5 (http://dx.doi.org/10.1289/ehp.1205736)].

## Discussion

An increased prevalence of gestational diabetes with increasing air pollution exposure was observed in an area with annual average measurements (2004–2005) of around 20 µg/m^3^ at urban background sites and around 28 µg/m^3^ at traffic sites, which is well below current WHO air quality guidelines (NO_2_ annual average < 40 µg/m^3^; WHO 2006). Also, an association between air pollution exposure and preeclampsia was observed. The associations remained when we adjusted for confounders and performed analyses that were restricted to women who did not move during pregnancy, and women who were Nordic-born or first-time mothers. Positive associations with NO_x_ exposure were evident in both urban and rural settings. For gestational diabetes and all cases of preeclampsia combined, associations increased monotonically with each quartile of NO_x_. Mild preeclampsia was significantly associated with all exposures above the first quartile, whereas severe preeclampsia was associated with the highest quartile of exposure only.

*Strengths and weaknesses*. The main strength of the present study is the large dataset from the Swedish Medical Birth Registry, comprising routinely assembled information from antenatal health care clinics covering almost all pregnancies in the region. Thus, the risk of selection bias was minimized, and well-known risk factors could be taken into account. Notably, almost all pregnant women were screened for glucose intolerance, and strict criteria were applied for the diagnosis of gestational diabetes and preeclampsia. Although effect estimates generally decreased after adjustment for covariates, the magnitude of changes was modest. As in all observational studies, there is a potential for bias due to confounding by unmeasured risk factors. However, risk factors such as family history of type 2 diabetes, polycystic ovary syndromes, and low own birth weight are not likely to act as confounders because they are not likely to be associated with the exposure. Another limitation is missing data, especially for BMI and smoking, which could be a potential source of bias.

A limitation was the lack of individual-level information on socioeconomic status, which could be associated with the outcomes and with the exposures. To address this, we performed separate analyses limited to Nordic-born women to achieve a more homogenous study group, because previous studies in Scania have reported associations between country of origin, air pollution, and socioeconomic status in the study area (Chaix et al. 2006; Malmqvist et al. 2011; Stroh et al. 2005). Previous studies using Swedish Medical Birth Registry data have reported that associations between maternal education (a proxy measure of socioeconomic status) and outcomes such as low birth weight and preterm birth could be explained almost entirely by a higher prevalence of smoking among women with lower levels of education (Källén 1999). Thus, we believe that unmeasured confounding by factors related to socioeconomic status was unlikely to have caused the associations observed.

One strength of the study is the individually modeled NO_x_ exposure estimates. Modeled and measured outdoor data show a good correspondence (Stroh et al. 2012). A spatial resolution of 500 × 500 m is appropriately accurate for aggregated monthly means in this study area (Stroh et al. 2007). The resolution should accordingly be reasonable for trimester means as well, because temporal aggregation allowed lower spatial resolution. Road traffic data have higher spatial resolution, but traffic density can be misclassified because traffic density measurements are not always performed on a yearly basis. We classified traffic exposure based on roads with the highest traffic density within a 200-m radius only. Regardless, the chosen proxies do not reflect the true individual exposure because a large proportion of traffic exposure, particularly for adults, takes place during commuting and at the workplace (Ritz et al. 2007).

An inherent problem is that the NO_x_ and traffic exposure categories were strongly correlated with urban/non-urban contrasts. In the subcohort of Nordic-born women, there was an urban/rural contrast with higher prevalence of exposure in the two main cities. However, contrasts within urban as well as non-urban areas were also observed, with higher prevalence with higher exposure to NO_x_. Proposing that the results obtained in this study reach beyond urban/nonurban contrasts would consequently suggest a true association between air pollution and gestational diabetes and preeclampsia.

*Previous studies*. To our knowledge, only a few research groups have investigated possible associations between gestational complications and air pollution (Lee et al. 2012; Rudra et al. 2011; van den Hooven et al. 2009, 2011; Vinikoor-Imler et al. 2012; Wu et al. 2009, 2011). Lee et al. (2012) reported positive associations between preeclampsia and model-based estimates of first-trimester PM_2.5_ and O_3_, but not PM_10_, in a cohort of > 34,000 births, whereas Vinikoor-Imler et al. (2012) reported associations between gestational hypertension (including preeclampsia) and PM_2.5_ and PM_10_ in a study of > 200,000 births. Wu et al. (2009) reported positive associations between preeclampsia and NO_x_ and PM_2.5_. Wu et al. (2011) found an association between preeclampsia and a variety of measures of air pollution exposure (measured as CO, NO, NO_2_, NO_x_, and PM_2.5_) regardless of the exposure assessment method employed; however, estimated effect depended on temporal and spatial variation in the models. Rudra et al. (2011) reported no associations between exposure and preeclampsia, but the study was relatively small. Van den Hooven et al. (2009) did not find an association with traffic density and gestational complications, but in a latter study (van den Hooven et al. 2011) they found an association between gestational hypertension and exposure to NO_2_ and PM_10_. On the relationship between exposure and preeclampsia, however, their results were inconclusive.

*Plausibility and comments, from a medical point of view*. The biological plausibility of air pollution (specifically fine particulate matter) affecting health has been discussed by Pope and Dockery (2006), focusing on exposure effects on blood pressure (Brook 2008) and the cardiovascular system (Brook 2008), aspects of substantial relevance to health during pregnancy (Yoder et al. 2009). The hypothesized biological pathways comprise systemic inflammation and placental oxidative stress (Redman and Sargent 2003; Sibai et al. 2005; Terzano et al. 2010). Previous studies have shown a decreased risk of preeclampsia in relation to tobacco smoking (Sibai et al. 2005). However, a possible explanation could be that air pollution from traffic and tobacco smoking do have some chemical components in common, whereas other components differ.

To put the findings of our study into perspective, the estimated effects of air pollution on pregnancy should be compared with the estimated effects of well-known risk factors. Among women born in Nordic countries, the association between the highest versus lowest quartile of NO_x_ exposure and gestational diabetes was comparable to the estimated effect of being overweight, but weaker than the estimated effect of being obese. Similarly, the association between high NO_x_ exposure and preeclampsia was similar to the estimated effects of having gestational diabetes, being overweight, or being > 35 years of age.

Gestational complications, such as gestational diabetes and preeclampsia, have implications not only for fetal morbidity and mortality, but also for maternal health. It has been estimated that one of three women with gestational diabetes will eventually develop type 2 diabetes (Linné et al. 2002). If the association between air pollution and pregnancy complications represents a causal relation, it would have profound implications for public health, particularly in countries with limited maternal health care.

## Conclusion

Statistically significant associations between NO_x_ exposure and gestational diabetes and preeclampsia were observed in a population exposed to air pollution at levels below current air quality guidelines.

## Supplemental Material

(266 KB) PDF
