# Supplementary material for: Gestational Diabetes and Preeclampsia in Association with Air Pollution at Levels below Current Air Quality Guidelines
Source: Environ Health Perspect. 2013 Jan 16;121(4):488–93. doi: 10.1289/ehp.1205736 (PMC3620758; doi:10.1289/ehp.1205736)
Supplement: (266 KB) PDF [file ehp.1205736.s001.pdf]

Supplemental Material

Gestational Diabetes and Preeclampsia in Association with Air Pollution at Levels below Current Air Quality Guidelines

Ebba Malmqvist, Kristina Jakobsson, Håkan Tinnerberg, Anna Rignell-Hydbom, Lars Rylander

Division of Occupational and Environmental Medicine, Lund University, Lund, Sweden

Table of Contents

Table S1. Background information of analyses of NO_x exposure during third trimester...........2
Table S2. Odds ratio for gestational diabetes in relation to NO_x (in quartiles) and traffic density (measured as vehicles/min)..3
Table S3: Odds ratio for gestational diabetes outcome during 1st trimester in relation to NO_x exposure...3
Table S4. ORs of outcomes in Nordic-born women, in relation to urban/non-urban contrasts, and contrasts within urban and non-urban areas...4
Table S5. ORs and 95% CI for Nordic born women obtained from multivariate logistic regression looking at actual exposure and known risk factors. 5
Table S6. Odds ratio of preeclampsia in relation to NO_x (in quartiles and measured in $\mu g/m^3$) and traffic density (measured as vehicles/min) 6
Table S7: Odds ratio for preeclampsia outcome during 1st trimester and 2nd trimester in relation to NO_x exposure 7

Table S1. Background information of analyses of NO_x exposure during third trimester

Potential confounders	**1st quartile of NO_x** 2.5-8.9 µg/m^3 N (%)	**2nd quartile of NO_x** 9.0-14.1 µg/m^3 N (%)	**3rd quartile of NO_x** 14.2-22.6 µg/m^3 N (%)	**4th quartile of NO_x** >22.7 µg/m^3 N (%)
**Maternal Age**				
<25	2786 (13.5)	2377 (12.1)	2537 (13.2)	2929 (17.2)
25-30	7033 (34.2)	6368 (32.3)	5719 (29.7)	5627 (33.1)
30-35	7084 (34.4)	7322 (37.1)	7104 (36.9)	5519 (32.5)
≥35	3677 (17.9)	3659 (18.5)	3879 (20.2)	2905 (17.1)
**Country of Origin**				
Sweden	18395 (90.9)	16608 (85.6)	14013 (74.2)	10593 (63.4)
Other Nordic	332 (1.6)	336 (1.7)	331 (1.8)	301 (1.8)
Other Western	192 (0.9)	259 (1.3)	313 (1.7)	232 (1.4)
Eastern Europe	643 (3.2)	966 (5.0)	1603 (8.5)	1823 (10.9)
Africa Sub Sahara	93 (0.5)	98 (0.5)	241 (1.3)	375 (2.2)
Middle East/North Africa	220 (1.1)	568 (2.9)	1760 (9.3)	2615 (15.6)
Asia	262 (1.3)	433 (2.2)	425 (2.3)	529 (3.2)
South/Central America	97 (0.5)	128 (0.7)	201 (1.1)	252 (1.5)
**Parity**				
1	8216 (40.2)	8749 (44.6)	9478 (49.4)	9289 (54.8)
2	7563 (37.0)	7241 (36.9)	6276 (32.7)	4566 (26.9)
≥3	4649 (22.8)	3636 (18.5)	3414 (17.8)	3104 (18.3)
**BMI**				
<18.5	532 (2.6)	739 (3.7)	750 (3.9)	711 (4.2)
18.5-24.9	7362 (35.8)	7699 (39.0)	7868 (40.9)	6982 (41.1)
25-29.9	5041 (24.5)	4288 (21.7)	4062 (21.1)	3534 (20.8)
≥30	2591 (12.6)	1845 (9.4)	1581 (8.2)	1462 (8.6)
Missing	5054 (24.6)	5155 (26.1)	4978 (25.9)	4291 (25.3)
**Smoking (cig/day)**				
0	16643 (80.9)	16242 (82.3)	15858 (82.4)	13765 (81.1)
1-9	1739 (8.4)	1537 (7.8)	1432 (7.4)	1413 (8.3)
>10	837 (4.1)	646 (3.3)	592 (3.1)	716 (4.2)
Missing	1361 (6.6)	1301 (6.6)	1357 (7.1)	1086 (6.4)
**Diabetes type 1**	93 (0.5)	105 (0.5)	95 (0.5)	94 (0.6)
**Chronic hypertension**	64 (0.3)	81 (0.4)	93 (0.5)	86 (0.5)
**Changed residency**	2339 (11.4)	2597 (13.2)	2469 (12.8)	1734 (10.2)
**Birth Year**				
1999	2255 (11.0)	2517 (12.8)	2310 (12.0)	3227 (19.0)
2000	2481 (12.1)	2618 (13.3)	2589 (13.5)	2861 (16.8)
2001	2529 (12.3)	2711 (13.7)	2586 (13.4)	2991 (17.6)
2002	3195 (15.5)	2903 (14.7)	3001 (15.6)	2347 (13.8)
2003	3033 (14.7)	2958 (15.0)	2699 (14.0)	2469 (14.5)
2004	3285 (16.0)	2936 (14.9)	3016 (15.7)	1836 (10.8)
2005	3802 (18.5)	3083 (15.6)	3038 (15.8)	1249 (7.4)
**Gestational diabetes**	282 (1.4)	341 (1.7)	448 (2.3)	464 (2.7)

Table S2. Odds ratio for gestational diabetes in relation to NO_x (in quartiles) and traffic density (measured as vehicles/min)

Gestational diabetes	Adjusted Non movers OR (95% CI)	Adjusted Nordic born OR (95% CI	Adjusted Primiparae OR (95% CI
**NO_x-quartiles ($\mu g/m^3$)**			
Q1=2.5-8.9	1.00 (ref)	1.00 (ref)	1.00 (ref)
Q2=9.0-14.1	1.24 (1.02, 1.51)	1.33 (1.09, 1.63)	1.34 (1.00, 1.81)
Q3=14.2-22.6	1.66 (1.38, 2.00)	1.57 (1.28, 1.93)	1.66 (1.25, 2.20)
Q4=>22.7	1.80 (1.49, 2.19)	1.56 (1.24, 1.95)	1.53 (1.14, 2.05)
**Traffic density within 200m**			
no road	1.00 (ref)	1.00 (ref)	1.00 (ref)
<2	0.89 (0.73, 1.07)	0.83 (0.68, 1.01)	0.81 (0.60, 1.08)
2-5	0.92 (0.77, 1.11)	0.95 (0.77, 1.16)	0.92 (0.70, 1.21)
5-10	1.22 (0.99, 1.49)	1.01 (0.77, 1.31)	0.97 (0.71, 1.32)
>10	1.29 (1.05, 1.60)	1.17 (0.90, 1.53)	0.91 (0.67, 1.25)

Gestational diabetes model adjusting for parity, BMI, maternal age, calendar year and country of origin and exposure for second trimester.

Table S3: Odds ratio for gestational diabetes outcome during 1st trimester in relation to NO_x exposure

NO_X ($\mu g/m^3$)	Gestational diabetes			
	Crude		Adjusted*	
	OR	95% CI	OR	95% CI
**2.5-8.9**	1.00	(Ref)	1.00	(Ref)
**9.0-14.1**	1.32	1.10 – 1.59	1.27	1.05 – 1.53
**14.2-22.6**	1.73	1.45 – 2.05	1.48	1.23 – 1.77
**>22.7**	2.05	1.73 – 2.42	1.65	1.38 – 1.99

*Model adjusting for parity BMI, maternal age, calendar year and country of origin

Table S4. ORs of outcomes in Nordic-born women, in relation to urban/non-urban contrasts, and contrasts within urban and non-urban areas

Exposure	Gestational Diabetes Adjusted OR (95% CI)	Preeclampsia Adjusted OR (95% CI)	Preeclampsia mild Adjusted OR (95% CI)	Preeclampsia severe Adjusted OR (95% CI)
**Urban/non-urban**				
Outside Malmö/Helsingborg	1.00 (ref)	1.00 (ref)	1.00 (ref)	1.00 (ref)
Within Malmö/Helsingborg	1.20 (0.99, 1.46)	1.40 (1.27, 1.54)	1.43 (1.28, 1.60)	1.32 (1.08, 1.62)
**Urban contrast**				
below mean NO_x 21.2µg/m^3	1.00 (ref)	1.00 (ref)	1.00 (ref)	1.00 (ref)
above mean NO_x 21.2µg/m^3	1.15 (0.98, 1.34)	1.27 (1.13, 1.44)	1.43 (1.28, 1.60)	1.32 (0.99, 1.46)
**Non-urban contrast**				
below mean NO_x 8.9µg/m^3	1.00 (ref)	1.00 (ref)	1.00 (ref)	1.00 (ref)
above mean NO_x 8.9µg/m^3	1.37 (1.18, 1.60)	1.37 (1.24, 1.51)	1.43 (1.27, 1.60)	1.18 (0.95, 1.45)

Gestational diabetes model adjusting for parity, BMI, maternal age, calendar year and country of origin and exposure for second trimester.

Preeclampsia models adjusting for gestational diabetes, type 1 diabetes, smoking, parity, BMI, country of origin, calendar year and maternal age and exposure for third trimester.

Table S5. ORs and 95% CI for Nordic born women obtained from multivariate logistic regression looking at actual exposure and known risk factors.

**Potential risk factors**	**Gestational Diabetes** N=60520 OR (95% CI)	**Preeclampsia** N=60341 OR (95% CI	**Mild preeclampsia** N=60341 OR (95% CI	**Severe Preeclampsia** N=60341 OR (95% CI
**Gestational diabetes**	*	1.90(1.42, 2.55)	1.81(1.31, 2.51)	2.26 (1.28, 4.00)
**Diabetes type 1**	*	2.70 (1.76,4.15)	2.56 (1.58, 4.13)	2.61 (1.18, 5.81)
**Maternal Age**				
<25	0.74 (0.54, 1.02)	1.12 (0.96, 1.31)	1.14 (0.96, 1.35)	0.98 (0.69, 1.39)
25-30	1.00 (ref)	1.00 (ref)	1.00 (ref)	1.00 (ref)
30-35	1.35 (1.11, 1.63)	1.04 (0.93, 1.17)	0.99 (0.87, 1.13)	1.31 (1.02, 1.67)
≥35	2.32 (1.89, 2.84)	1.20 (1.03, 1.39)	1.14 (0.97, 1.34)	1.43 (1.05, 1.95)
**Smoking (cig/day)**				
0	*	1.00 (ref)	1.00 (ref)	1.00 (ref)
1-9	*	0.65 (0.52, 0.79)	0.67 (0.53, 0.84)	0.57 (0.34, 0.93)
>10	*	0.56 (0.40, 0.77)	0.56 (0.39, 0.81)	0.54 (0.26, 1.16)
**Parity**				
1	1.07 (0.90, 1.26)	2.67 (2.35, 3.00)	2.62 (2.28, 3.01)	2.68 (2.07, 3.47)
2	1.00 (ref)	1.00 (ref)	1.00 (ref)	1.00 (ref)
≥3	0.96 (0.77, 1.18)	0.86 (0.70, 1.04)	0.91 (0.74, 1.13)	0.66 (0.42, 1.04)
**BMI**				
<18.5	1.73 (1.04, 2.88)	0.63 (0.39, 1.03)	0.47 (0.25, 0.88)	1.17 (0.55, 2.52)
18.5-24.9	1.00 (ref)	1.00 (ref)	1.00 (ref)	1.00 (ref)
25-29.9	1.87 (1.55, 2.25)	1.85 (1.64, 2.09)	1.93 (1.69, 2.21)	1.53 (1.19, 1.98)
≥30	4.05 (3.34, 4.91)	3.75 (3.28, 4.28)	4.07 (3.52, 4.70)	2.28 (1.68, 3.10)
**NO_x ($\mu g/m^3$)**				
Q1=2.5-8.9	1.00 (ref)	1.00 (ref)	1.00 (ref)	1.00 (ref)
Q2=9.0-14.1	1.33 (1.09, 1.63)	1.24 (1.08, 1.41)	1.34 (1.15, 1.55)	0.90 (0.67, 1.20)
Q3=14.2-22.6	1.57 (1.28, 1.93)	1.33 (1.16, 1.53)	1.43 (1.22, 1.66)	1.04 (0.78, 1.39)
Q4=>22.7	1.56 (1.24, 1.95)	1.51 (1.31, 1.74)	1.53 (1.30, 1.80)	1.39 (1.04, 1.85)

Gestational diabetes models adjusting for parity, BMI, maternal age, calendar year and country of origin and exposure for second trimester.

Preeclampsia models adjusting for gestational diabetes, type 1 diabetes, smoking, parity, BMI, country of origin, calendar year and maternal age and exposure for third trimester.

* not a known risk factor

Table S6. Odds ratio of preeclampsia in relation to NO_x (in quartiles and measured in µg/m^3) and traffic density (measured as vehicles/min)

Outcome/exposure	Adjusted Non movers OR (95% CI)	Adjusted Nordic born OR (95% CI)	Adjusted Primiparae OR (95% CI)
**Preeclampsia/NO_x quartiles**			
Q1=2.5-8.9	1.00 (ref)	1.00 (ref)	1.00 (ref)
Q2=9.0-14.1	1.26 (1.09, 1.44)	1.24 (1.08, 1.41)	1.34 (1.14, 1.58)
Q3=14.2-22.6	1.29 (1.12, 1.49)	1.33 (1.16, 1.53)	1.44 (1.22, 1.69)
Q4=>22.7	1.48 (1.27, 1.70)	1.51 (1.31. 1.74)	1.61 (1.37, 1.90)
**Preeclampsia mild/NO_x-quartiles**			
Q1=2.5-8.9	1.00 (ref)	1.00 (ref)	1.00 (ref)
Q2=9.0-14.1	1.35 (1.15, 1.58)	1.34 (1.16, 1.56)	1.39 (1.16, 1.67)
Q3=14.2-22.6	1.39 (1.18, 1.63)	1.42 (1.22, 1.66)	1.53 (1.28, 1.84)
Q4=>22.7	1.48 (1.25, 1.75)	1.54 (1.30, 1.81)	1.60 (1.32, 1.93)
**Preeclampsia severe/NO_x-quartiles**			
Q1=2.5-8.9	1.00 (ref)	1.00 (ref)	1.00 (ref)
Q2=9.0-14.1	0.92 (0.68, 1.25)	0.89 (0.66, 1.20)	1.14 (0.81, 1.63)
Q3=14.2-22.6	0.93 (0.68, 1.27)	1.01 (0.75, 1.37)	1.07 (0.75, 1.53)
Q4=>22.7	1.41(1.05, 1.89)	1.40(1.04, 1.88)	1.63(1.16, 2.28)
**Preeclampsia/ Traffic Density 200m**			
**no road**	1.00 (ref)	1.00 (ref)	1.00 (ref)
<2	1.08 (0.94, 1.25)	1.12 (0.97, 1.28)	1.27 (1.07, 1.51)
2-5	1.01 (0.87, 1.16)	1.03 (0.90, 1.19)	1.19 (1.00, 1.41)
5-10	1.13 (0.96, 1.35)	1.20 (1.01, 1.43)	1.29 (1.06, 1.57)
>10	1.01 (0.84, 1.22)	1.12 (0.93, 1.34)	1.32 (1.09, 1.60)
**Preeclampsia mild/Traffic Density 200 m**			
**no road**	1.00 (ref)	1.00 (ref)	1.00 (ref)
<2	1.03 (0.88, 1.20)	1.06 (0.90, 1.23)	1.20 (0.99, 1.46)
2-5	0.99 (0.84, 1.16)	1.03(0.88, 1.20)	1.15(0.95, 1.39)
5-10	1.14 (0.94, 1.38)	1.20 (0.99, 1.46)	1.31 (1.05, 1.63)
>10	0.96 (0.78, 1.19)	1.05 (0.85, 1.29)	1.27 (1.02, 1.58)
**Preeclampsia severe/Traffic Density 200 m**			
**no road**	1.00 (ref)	1.00 (ref)	1.00 (ref)
<2	1.28 (0.95, 1.72)	1.31 (0.97, 1.77)	1.49 (1.03, 2.14)
2-5	1.08 (0.79, 1.47)	1.08 (0.78, 1.47)	1.35 (0.94, 1.93)
5-10	1.03 (0.70, 1.53)	1.15 (0.78, 1.69)	1.10 (0.71, 1.72)
>10	1.17 (0.80, 1.73)	1.29 (0.88, 1.89)	1.40 (0.92, 2.12)

Preeclampsia models adjusting for gestational diabetes, type 1 diabetes, smoking, parity, BMI, country of origin, calendar year and maternal age.

Table S7: Odds ratio for preeclampsia outcome during 1st trimester and 2nd trimester in relation to NO_x exposure

NO_X (µg/m³)	Preeclampsia (all) 1st trimester			
	Crude		Adjusted*	
	OR	95% CI	OR	95% CI
**2.5-8.9**	1.00	(Ref)	1.00	(Ref)
**9.0-14.1**	1.24	1.10 – 1.41	1.29	1.13 – 1.47
**14.2-22.6**	1.20	1.06 – 1.36	1.26	1.10 – 1.44
**>22.7**	1.31	1.15 – 1.48	1.43	1.25 – 1.63
**NO_X (µg/m³)**	**Preeclampsia (mild) 1st trimester**			
	Crude		Adjusted*	
	OR	95% CI	OR	95% CI
**2.5-8.9**	1.00	(Ref)	1.00	(Ref)
**9.0-14.1**	1.34	1.16 – 1.54	1.42	1.22 – 1.64
**14.2-22.6**	1.32	1.14 – 1.52	1.41	1.22 – 1.64
**>22.7**	1.31	1.14 – 1.52	1.47	1.26 – 1.71
**NO_X (µg/m³)**	**Preeclampsia (severe) 1st trimester**			
	Crude		Adjusted*	
	OR	95% CI	OR	95% CI
**2.5-8.9**	1.00	(Ref)	1.00 (Ref)	(Ref)
**9.0-14.1**	0.98	0.76 – 1.26	0.95	0.72 – 1.24
**14.2-22.6**	0.95	0.73 – 1.22	0.87	0.66 – 1.15
**>22.7**	1.27	1.00 – 1.62	1.30	1.00 – 1.69
**NO_X (µg/m³)**	**Preeclampsia (all) 2nd trimester**			
	Crude		Adjusted*	
	OR	95% CI	OR	95% CI
**2.5-8.9**	1.00	(Ref)	1.00	(Ref)
**9.0-14.1**	1.24	1.09 – 1.40	1.30	1.14 – 1.48
**14.2-22.6**	1.22	1.08 – 1.39	1.30	1.14 – 1.48
**>22.7**	1.34	1.18 – 1.52	1.50	1.31 – 1.71
**NO_X (µg/m³)**	**Preeclampsia (mild) 2nd trimester**			
	Crude		Adjusted*	
	OR	95% CI	OR	95% CI
**2.5-8.9**	1.00	(Ref)	1.00	(Ref)
**9.0-14.1**	1.37	1.19 – 1.58	1.46	1.26 – 1.69
**14.2-22.6**	1.30	1.12 – 1.50	1.41	1.21 – 1.64
**>22.7**	1.38	1.20 – 1.59	1.59	1.36 – 1.86
**NO_X (µg/m³)**	**Preeclampsia (severe) 2nd trimester**			
	Crude		Adjusted*	
	OR	95% CI	OR	95% CI
**2.5-8.9**	1.00	(Ref)	1.00	(Ref)
**9.0-14.1**	0.91	0.70 – 1.17	0.87	0.66 – 1.15
**14.2-22.6**	1.07	0.84 – 1.37	1.02	0.78 – 1.33
**>22.7**	1.23	0.97 – 1.56	1.24	0.95 – 1.62

*Model adjusting for gestational diabetes, type 1 diabetes, smoking, parity BMI, country of origin, calendar year and maternal age
